# Dimethyl disulfide (DMDS) as an effective soil fumigant against nematodes in China

**DOI:** 10.1371/journal.pone.0224456

**Published:** 2019-10-28

**Authors:** Dongdong Yan, Aocheng Cao, Qiuxia Wang, Yuan Li, Ouyang Canbin, Meixia Guo, Xiaoqin Guo

**Affiliations:** Institute of Plant Protection, Chinese Academy of Agricultural Sciences, Beijing, China; Peking University, CHINA

## Abstract

Root-knot nematode is an important soil pest in horticulture crops and constrains the protected cultivation development after methyl bromide (MB) was phased out in China. Dimethyl disulfide (DMDS) exhibits excellent efficacy against nematodes. Laboratory experiments and field trials were set up to clarify DMDS dose, efficacy, and yield. A dose-response experiment using three methods showed that DMDS presented high efficacy against the nematode *Meloidogyne incongnita*. The LC_50_ values of direct fumigation activity in the dessicator method were 0.086 and 0.070 mg L^-1^ for DMDS and 1,3-D, 29.865 and 18.851 mg L^-1^ for DMDS and 1,3-D of direct contact activity in the small tube method, 6.438 and 3.061 mg L^-1^ for DMDS and 1,3-D of soil fumigation activity in the soil fumigation method, respectively. The field trials indicated that DMDS showed an excellent efficacy of 80%−94% on root-knot nematode applied at 10−100 g m^−2^ on tomato in Tongzhou, Beijing. The crop yields showed no significant difference after applying 10–80 g m^-2^ DMDS. Results indicate that DMDS applied at 10 g m^-2^ for controlling root-knot nematode in Beijing is cost effective. In conclusion, DMDS is an excellent soil fumigant that can be used for controlling root-knot nematode and can be an potential novel alternative to MB in China.

## Introduction

Protected cultivation of high-value crops are important techniques used to increase farmers’ income and modernize traditional agriculture. These techniques have become increasingly important in China’s economy and thus are expected to continue booming. The area for vegetable cultivation in China is 24,549,160 ha in 2015 [1]. However, the occurrence of root-knot nematode (*Meloidogyne* spp.) is becoming more severe, causing farmers to suffer huge losses. Crop yield and quality are usually decreased significantly after 3–5 years of cultivation without rotation. The yield losses are normally 20% to 40%, though they may reach more than 60% or total yield losses [2].

Methyl bromide (MB) is a highly effective fumigant used to control various soilborne pests for approximately past 50 years. Farmers gained good profits from using MB, which engendered their dependence on this chemical. Although useful for pest management [3], MB was included on the list of substances that deplete the stratospheric ozone layer under the Montreal Protocol, and its registration in cucumber and tomato was cancelled in China in 2011. Therefore, economical, effective, and feasible alternatives to MB must be found to reduce the real losses caused by the phasing out of MB in China.

Dimethyl disulfide (DMDS) is a sulfide-based volatile compound that has no ozone depletion potential. DMDS exhibits great insecticidal and fungicidal activities in vitro study[4, 5]. And DMDS is highly efficient in controlling many soilborne diseases and nematodes of numerous crops, such as tomato[6], cucumber[7], strawberry[8] and other crops [9, 10]. This fumigant also provides good control on yellow nutsedge [11, 12]. The efficacy of DMDS can be improved when combined with virtually impermeable films or totally impermeable films [13].

Aim of the present study was to evaluate the efficacy of DMDS to control nematodes and its feasibility as an MB alternative in soil fumigation. The suitable DMDS dosage for practical use in China was also identified.

## Materials and methods

### Laboratory dose-response experiments

Three methods were used to identify the dose response curve on chemicals direct contact and fumigation activity and soil fumigation activity: small tube, desiccator, and soil fumigation [14]. The small tube method was applied to evaluate the contact toxicity of chemicals. A nematode egg mass was isolated from tomato knot roots collected from Tongzhou, Beijing. The egg mass was hatched at 28°C in an incubator. After 3 days, the hatched eggs, which contained about 150 nematodes in 0.5 mL of water, was transferred to a 1.5 mL small tube. 1,3-D (95% 1,3-D technical concentrate, provided by Beijing Zhongzhikehua Agricultural Technology Co. Ltd.) and DMDS (98% DMDS technical concentrate provided by Linhai Jianxin Chemical Co. Ltd.) were diluted into the following concentrations: 2.5, 5, 10, 20, 40 and 80 mg L^-1^. Distilled water was used as control. The number of dead nematodes was calculated after 24 h. All dosages were repeated three times.

The desiccator method was applied to evaluate the direct fumigation toxicity of chemicals toward nematodes. A 2.5 L desiccator was prepared for the experiment. The nematode suspension, which contained about 150 nematodes in 100 μL of water, was transferred to a double concave slide glass. Each treatment was repeated six times. Exactly 5 mL of distilled water for moisture was poured into the bottom of the desiccator, and the double concave slide glass was placed inside. 1,3-D and DMDS were added to a small culture dish, and the final concentration was adjusted to 0.8, 0.4, 0.2, 0.1, and 0.05 mg L^−1^ in the desiccator. The desiccator was covered quickly and placed in an incubator at 28°C. The number of dead nematodes was calculated after 72 h.

The soil fumigation method was applied to evaluate the fumigation toxicity of chemicals in soil. Soil samples (300 g) heavily infected with nematodes were collected from Tongzhou and stored in a 500 mL wide-mouth bottle, and 1,3-D or DMDS was pipetted into the bottle. The final dosages of DMDS were 80, 40, 20, 10, 5, and 2.5 mg kg^−1^, and the dosages of 1,3-D were 40, 20, 10, 5, 2.5, and 1.15 mg kg^−1^. After 6 days of fumigation, 100 g of soil was collected for nematode analysis and 5 g of soil for pathogen analysis. *Fusarium* spp. and *Phytophthora* spp. populations were quantified as indicators of the relative efficacy of each treatment in controlling soilborne fungal pathogens. *Fusarium* spp. and *Phytophthora* spp. were isolated according to Komada’s method [15] and Masago’s method [16], respectively. Soil nematodes (*Meloidogyne* spp.) in soil were extracted based on the sugar-flotation-sieving method [17].

### Field experiments

Field experiments were conducted in 2011−2014 in Tongzhou and Fangshan, Beijing. The field studies were authorized by the Tongzhou Agricultural Science Institution and Fangshan Agricultural Science Institution (Beijing City), and no other specific permissions were required for the field site. The field studies did not involve any endangered or protected species. The conventional crop pattern is tomato−cucumber rotation system. Root-knot nematode has been severe due to consecutive planting without effective rotation. Detailed soil analytical data of different field sites are presented in Table 1.

**Table 1:** Basic physical and chemical properties of the experimental soil.

Soil	Ammonium (mg kg^−1^)	Nitrate (mg kg^−1^)	Available phosphorus (mg kg^−1^)	Available potassium (mg kg^−1^)	Organic matter (g kg^−1^)	pH	Water content (%)
2011 Tongzhou	3.3	118.5	349.9	335.1	30.5	7.3	17.4
2012 Tongzhou	13.9	205.2	347.5	632.6	38.2	7.4	18.2
2014 Tongzhou	16.4	423.4	387.5	790.0	40.4	6.3	15.9
2014 Fangshan	25.9	603.1	425.1	655.0	29.9	7.0	8.2

Field trials were conducted in greenhouses with areas within 800 m^2^. Each greenhouse was divided into completely randomized plots corresponding to different treatments: 60, 80, and 100 g m^-2^ DMDS; 50 g m^-2^ MB; 9 g m^-2^ 1,3-D and untreated control for 2011−2012 and 10, 20, 30, and 40 g m^-2^ DMDS; 30 g m^-2^ dazomet; 9 g m^-2^ 1,3-D and untreated control for 2014. Each treatment has four replications. The plot area was 24 m^2^. The crops were double-row planted with the density of 45,000 plants ha^-1^. All of the plots treated with DMDS, 1,3-D, dazomet, and MB were covered with polyethylene film (40 μm thickness) after fumigation. DMDS and 1,3-D were applied using hand injection, and MB was applied using hot gas. Dazomet was applied through soil incorporation. The covered film for fumigation was removed from all plots after about 7 days after fumigation. Normal cultivation techniques were used in all treatments. Information about the soil treatment and cultivation is listed in Table 2. The severity of root-knot disease was evaluated at the end of the trials. Twenty plants were picked from each plot, and the severity of root-knot disease were assessed. Marketable yields of cucumber or tomato were recorded at each harvest.

**Table 2:** Variety and growth calendar of the vegetables.

Year	Field Site	Crop/Variety	Seedling	Transplanting	Beginning of harvesting	Finish of the season
2011	Tongzhou	cucumber/ Zhongnong 16	Aug. 22, 2011	Sep. 5, 2011	Oct.14, 2011	Dec. 2, 2011
2012	Tongzhou	cucumber/ Zhongnong 16	Aug. 18, 2012	Aug. 28, 2012	Oct. 2, 2012	Dec. 3, 2012
2014	Tongzhou	cucumber/ Zhongnong 16	Aug. 16, 2014	Aug. 25, 2014	Sep. 29, 2014	Dec. 1, 2014
2014	Fangshan	Tomato/ Nongda 3	Jul. 20, 2014	Aug. 13, 2014	Oct. 27, 2014	Dec. 31, 2014

### Statistical analysis

#### Laboratory study

Nematode mortality was calculated using the following formula:

$$M=\frac{N_{1}}{N_{1}+N_{2}}\times 100,$$ (1)

where *M* is percentages of the nematode mortality (%), *N*_*1*_ is the number of dead nematodes, and *N*_*2*_ is the number of alive nematodes.

The efficacy on nematodes after fumigation was calculated using the following formula:

$$E_{n}=\frac{M_{1}-M_{2}}{1-M_{2}}\times 100,$$ (2)

where *En* is the efficacy on nematodes (%), *M*_*1*_ is percentages of the nematode mortality of treatments (%), and *M*_*2*_ is percentages of the nematode mortality of untreated control (%).

The observed efficacy on soil pathogens was calculated using the following formula:

$$E_{f}=\frac{P_{1}-P_{2}}{P_{1}}\times 100,$$ (3)

where *E*_*f*_ is the efficacy on fungi (%), *P*_*1*_ is the population density of untreated control, and *P*_*2*_ is the population density under the treatments.

The dose response relationship was analyzed by using the Probit method. LC_50_ and regression equation were calculated using DPS software(Zhejiang University, China), Probit analysis [18].

#### Field trials

The root galling index was assessed on 0 to 4 scale. 0 = no gall, 1 = 1–25%, 2 = 26–50%, 3 = 51–75%, and 4 = 76–100% roots galled [19]. The root galling index was calculated using the following formula described by McKinney [20]:

$$GI=\frac{\sum \left(n\cdot v\right)}{N\;\cdot X}\times 100,$$ (4)

where *GI* is the root galling index (%), *v* is the class value, *n* is the number of plants in each class, *N* is the number of observed plants, and *X* is highest value of the evaluation scale.

Data were subjected to variance analysis using DPS software (Zhejiang University, China). The percentage data for efficacy of galling index were transformed using arcsine transformation before variation analysis. The resulting data on yield were directly analyzed. Significant differences among means were compared by Fisher’s LSD test at *P* = 0.05.

## Results

### Effect of DMDS on soil pathogens in lab

As shown in Table 3, DMDS exhibited good bioactivity against nematodes. The LC_50_ values in the dessicator method were 0.086 and 0.070 mg L^-1^ for DMDS and 1,3-D, 29.865 and 18.851 mg L^-1^ for DMDS and 1,3-D in the small tube method, 6.438 and 3.061 mg L^-1^ for DMDS and 1,3-D in the soil fumigation method, respectively.

**Table 3:** Dose-response result of nematode on chemicals direct contact and fumigation activity and soil fumigation activity in small tube, desiccator, and soil fumigation methods.

Method	DMDS^Z^		1,3-D	
	LC_50_ (95% confidence limit mg L^−1^)	Regression equation	LC_50_ (95% confidence limit mg L^−1^)	Regression equation
Small tube	29.865 (23.118−38.581)	Y = 2.994+1.360x	18.851 (14.998−23.694)	Y = 3.072+1.512x
Desiccator	0.086 (0.067−0.110)	Y = 6.300+1.220x	0.070 (0.056−0.080)	Y = 6.700+1.445x
Soil fumigation	6.438 (4.605−9.003)	Y = 4.097+1.117x	3.061 (3.100−4.200)	Y = 4.150+1.526x

^Z^ Abbreviations: DMDS = dimethyl disulfide, 1,3-D = 1,3-dichloropropene.

The bioassay results of soil pathogens with the soil fumigation method in the laboratory are presented in Table 4. Dose-response results showed that the activity of DMDS against soil pathogens was lower than that of 1,3-D.

**Table 4:** Dose response result of soil pathogens to DMDS and 1,3-D with the soil fumigation method.

Soil pathogens	DMDS^Z^		1,3-D	
	LC_50_ (95% confidence limit mg L^−1^)	Regression equation	LC_50_ (95% confidence limit mg L^−1^)	Regression equation
*Phytophthora* spp.	2.670 (1.715–4.157)	Y = 4.410+1.383x	0.981 (0568–1.694)	Y = 5.007+0.804x
*Fusarium* spp.	3.901 (1.532–9.935)	Y = 4.239+1.288x	1.647 (0.946–20867)	Y = 4.797+0.938x

^Z^ Abbreviations: DMDS = dimethyl disulfide, 1,3-D = 1,3-dichloropropene.

### Effect of DMDS on root-knot disease and crop yields

The field experiments showed that DMDS at 60, 80, and 100 g m^−2^ effectively controlled cucumber nematodes in 2011 to 2012 (Table 5). No significant difference in nematode control and yield was observed among the treatments of DMDS, 1,3-D, and MB.

**Table 5:** Marketable yield of crop and galling index at harvest.

Field site and crop	Treatment ^y^	Dosage (g m^-2^)	Galling index of tomato and cucumber	Yield kg ha^-1^
2011 Tongzhou cucumber	DMDS	60	3.75 b ^z^	4838.41 ab
	DMDS	80	0 b	4410.69 b
	DMDS	100	0 b	4240.07 b
	MB	40	0 b	5495.86 a
	1,3-D	9	0 b	4253.11 b
	Untreated Control	-	49.38 a	2936.09 c
2012 Tongzhou cucumber	DMDS	60	0 b	7871.51 a
	DMDS	80	0 b	8044.55 a
	DMDS	100	0 b	8402.76 a
	MB	40	0 b	9022.01 a
	1,3-D	9	0 b	8757.63 a
	Untreated Control	-	72.50 a	5406.49 b
2014 Tongzhou cucumber	DMDS	10	17.50 b	4215.11 a
	DMDS	20	10.63 b	3732.32 ab
	DMDS	40	4.38 c	4041.56 a
	DMDS	80	0.63 c	4112.72 a
	DZ	30	13.75 b	3121.92 bc
	Untreated Control		66.25 a	2747.17 c
2014 Fangshan tomato	DMDS	10	76.25 ab	3642.87 a
	DMDS	20	62.50 ab	3674.98 a
	DMDS	40	48.75 bc	3915.23 a
	DMDS	80	18.75 c	3494.65 a
	1,3-D	9	61.25 b	3517.81 a
	Untreated Control	-	93.75 a	2362.91 b

^y^Abbreviations: 1,3-D = 1,3-dichloropropene, DMDS = dimethyl disulfide, MB = methyl bromide, DZ = Dazomet.

^z^Data are the means of three replications in the column. Means followed by the same letter are not significantly different (*P* = 0.05) according to the LSD test.

The galling index of cucumber was analyzed, and the results are displayed in Table 5. The root galling index of cucumber significantly decreased after treated with DMDS and MB at 40 g m^−2^ in the Tongzhou site. All DMDS and MB treatments significantly reduced the root galling index.

The yield of cucumber in 2011 to 2012 was significantly higher in all treated plots than untreated control treatment. No significant difference in yield was observed among the all treated plots. The highest yields were observed in MB treatment, and it was about twice as much as that in the untreated control.

**Fig 1:**
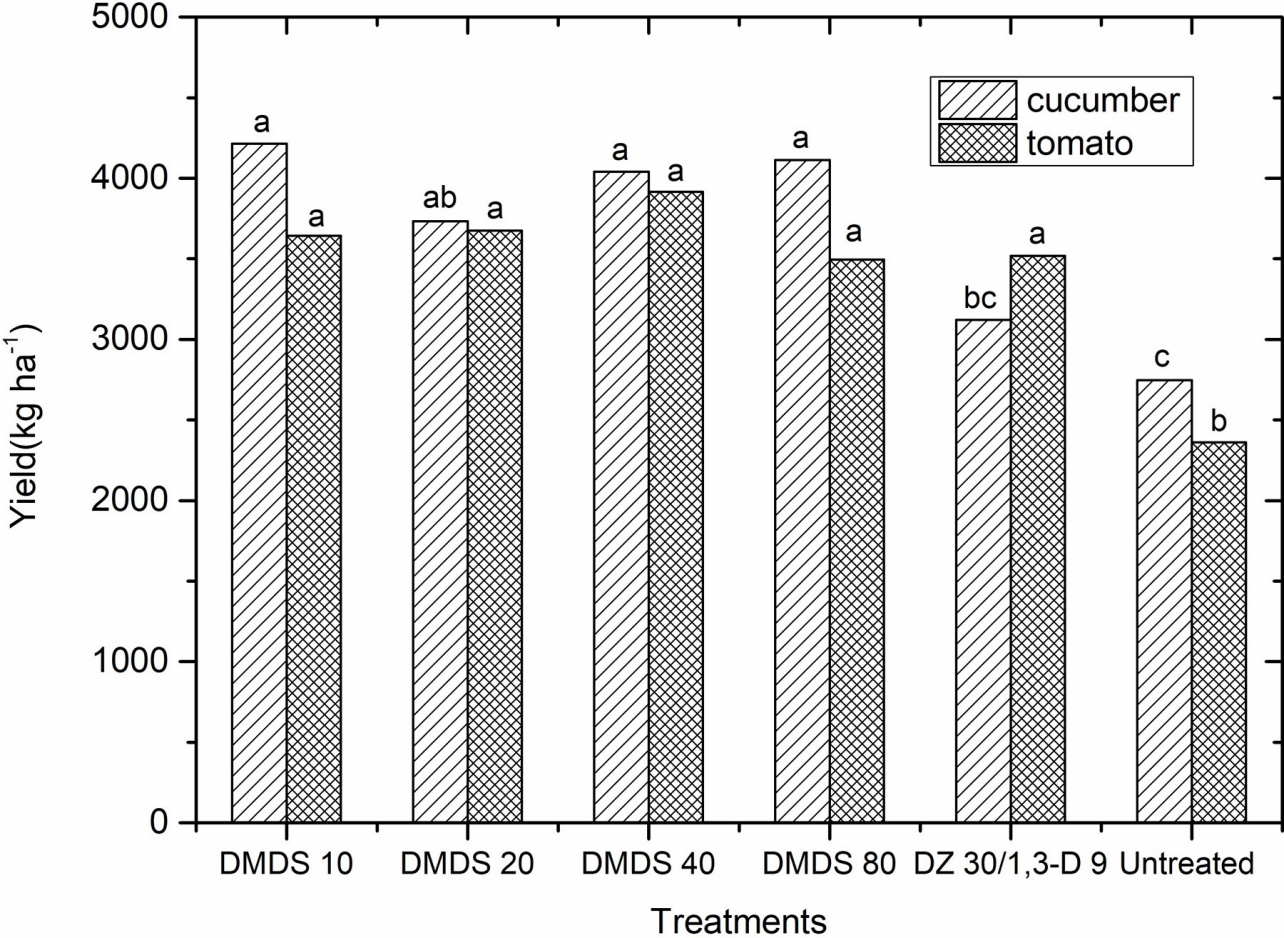
Cucumber and tomato yields after different fumigation treatments in the year 2014. Abbreviations: DMDS = dimethyl disulfide, 1,3-D = 1,3-dichloropropene, DZ = Dazomet, DMDS 10 = DMDS 10 g m^-2^ treatment, DZ 30 = DZ 30 g m^-2^ treatment of cucumber trials in the year 2014, 1,3-D 9 = 1,3-D 9 g m^-2^ treatment of tomato trials in the year 2014. Different letters indicate significant differences in each treatment group according to the LSD test.

Field trial results in 2014 showed that DMDS significantly reduced the root gall index of cucumber, even if DMDS was applied at low dosage. In tomato field, plants were seriously infected by root knot nematode at harvest time. The root gall index in untreated control reached up to 93.75%. DMDS applied at high dosage of 40, 80 g m^-2^ significantly reduced the root gall index of tomato. All DMDS fumigation treatments significantly increased the crop yields compared to untreated control in both cucumber and tomato fields (Fig 1), and the yields showed no significant difference among different application dosages.

## Discussion

Root-knot nematode has threatened the protected agriculture worldwide. With the phased-out of MB, new fumigants must be found to replace MB. 1,3-D is an excellent fumigant, but it poses environmental and health problems [21], which have greatly reduced its chances for registration in China. DMDS shows promising efficacy in controlling root-knot nematode with rates that can be as low as 10–20 g m^−2^. DMDS exhibited efficacy in controlling soil pathogens, such as *Fusarium* and *Phytophthora*, but its bioactivity was weaker than that of 1,3-D, which shows moderate efficacy in controlling soil pathogens. 1,3-D needs to be mixed with chloropicrin, a fumigant with high efficacy to control pathogens, for practical application as an alternative to MB. Same result was also observed by Cabrera, low dosages of DMDS were sufficient for nematode control, but less efficacious against soilborne pathogens[22]. High dosages of 60 g m^−2^ DMDS were also used to control *Fusarium* and *Verticillium* wilt disease[23, 24]. DMDS also needs to be mixed with chloropicrin for controlling nematodes and pathogens. Similarly, previous studies reported that the use of DMDS plus chloropicrin consistently improves early and total marketable strawberry yields due to successful soilborne fungi and nematode control [25, 26]. Combination use of DMDS plus dazomet and DMDS plus 1,3-D can also improve the control efficacy on root-knot nematode and soilborne fungi [7, 27].

Many novel fumigants and potential soil disinfestation technologies were developed to control soilborne disease besides DMDS. Allyl isothiocyanate (AITC) used as a soil fumigant effectively controlled major bacterial and fungal pathogens and root knot nematode[28]. Nonchemical methods of soil disinfestation include anaerobic soil disinfestation (ASD) [29] and soil biosolarization[30] were also widely used to control soilborne disease. Soil flame disinfestation, a promising non-chemical method, was also developed to control soilborne nematodes, fungal and bacterial pathogens in China[31]. Soilborne diseases caused damage to the crops throughout the whole growing season, biological control agent (BCA)[32] such as *Trichoderma harzianum* or Bacillus spp. and  plant growth-promoting rhizobacteria(PGPR) [33] were also applied combined with pre-plant soil treatment to control root and soilborne disease. BCA were also used for seed treatment and root drenching to improve plant vigor[34, 35]. Soil drenching with BCA in crop growing season also increase the control effect on soilborne disease.

DMDS is a technically and economically feasible soil fumigant for controlling root-knot nematode in China. The dose recommended is 10–20 g m^−2^ for controlling short-term crops, such as cucumber or tomato, in autumn.
